# 454 Pyrosequencing of Olive (*Olea europaea L*.) Transcriptome in Response to Salinity

**DOI:** 10.1371/journal.pone.0143000

**Published:** 2015-11-17

**Authors:** Christos Bazakos, Maria E. Manioudaki, Elena Sarropoulou, Thodhoraq Spano, Panagiotis Kalaitzis

**Affiliations:** 1 Department of Horticultural Genetics and Biotechnology, Mediterranean Agronomic Institute of Chania (MAICh), Crete, Greece; 2 Department of Horticulture, Aristotle University of Thessaloniki, Thessaloniki, Greece; 3 Institute of Marine Biology, Biotechnology and Aquaculture, Hellenic Centre for Marine Research, Heraklion, Crete, Greece; University of Minho, PORTUGAL

## Abstract

Olive (*Olea europaea* L.) is one of the most important crops in the Mediterranean region. The expansion of cultivation in areas irrigated with low quality and saline water has negative effects on growth and productivity however the investigation of the molecular basis of salt tolerance in olive trees has been only recently initiated. To this end, we investigated the molecular response of cultivar Kalamon to salinity stress using next-generation sequencing technology to explore the transcriptome profile of olive leaves and roots and identify differentially expressed genes that are related to salt tolerance response. Out of 291,958 obtained trimmed reads, 28,270 unique transcripts were identified of which 35% are annotated, a percentage that is comparable to similar reports on non-model plants. Among the 1,624 clusters in roots that comprise more than one read, 24 were differentially expressed comprising 9 down- and 15 up-regulated genes. Respectively, inleaves, among the 2,642 clusters, 70 were identified as differentially expressed, with 14 down- and 56 up-regulated genes. Using next-generation sequencing technology we were able to identify salt-response-related transcripts. Furthermore we provide an annotated transcriptome of olive as well as expression data, which are both significant tools for further molecular studies in olive.

## Introduction

Olive is one of the most important crops of the Mediterranean basin, an area where most of the total world olive oil is produced. The continuous expansion of olive cultivation in lands irrigated with low quality, mostly saline water poses serious threats to plant productivity.

Sodium chloride (NaCl) is the most soluble and widespread salt that negatively affects olive shoot growth [1–2], fruit productivity [3], alters photosynthesis [4–5] and causes morphological changes in leaves [6]. Although the tolerance of olives to NaCl is believed to be intermediate [7] extended genetic diversity on abiotic stress tolerance has been detected within olive germplasm [8–9]. Tolerance might be attributed to certain mechanisms such as retention of Na^+^ and Cl^-^ ions by the root and stem or ion exclusion due to K^+^ selectivity instead of Na^+^ [6]. Another mechanism is compartmentation of toxic ions through restriction of entrance of toxic ions such as Na^+^ or Cl^-^ into vacuoles [6].

Our knowledge on the response of olive trees to salinity is restricted to physiological level where numerous studies are available [6]. A recent study on the salt stress response of two cultivars, one tolerant and one sensitive, resulted in the identification of transcription factors which might play a significant role in this response using microarrays analysis [10]. However, the molecular basis of salt tolerance in olive has not been investigated thoroughly. The current lack of *Olea europaea* reference transcriptome sequence creates difficulties towards the identification and characterization of regulatory genes [11]. High-throughput transcriptome sequencing is a rapid, efficient and attractive alternative to microarrays approaches for gene expression studies [12–13]. Deep sequencing-based approaches have the potential to overcome the limitations of microarrays and provide the advantage of detecting transcriptome dynamics across different tissues and/or conditions [14–15]. Several studies have described the use of 454 Life Sciences (Roche) sequencing [16] as an efficient tool to examine mRNA expression levels [13,17–19]. Comparative deep sequencing studies of plant transcriptomes have identified gene expression alterations in response to tissue, genotype or physiological changes in model or non-model species such as maize [20], chestnut [21], grapevine [22], eucalyptus [23], waterhemp [24] and recently, in olive tree [25, 26, 27].

The response of a salt-tolerant cultivar, cv Kalamon [9], in comparison to the salt-sensitive, cv Chondrolia Chalkidikis, under a 45- and 90-day NaCl (120 mM) treatment, revealed the existence of a coordinated effort of transcript expression regulation [10]. In the present study, we used 454 GS FLX pyrosequencing platform (IMBBC-HCMR NGS Platform) to investigate the molecular response of the salt-tolerant cv Kalamon in order to characterize the transcriptome profile of olive roots and leaves and to identify differentially expressed transcripts that are related to salt tolerance response. The major advantage of 454-pyrosequencing platform for transcriptomic studies of non-model plants compared to other NGS platforms is the read length [19]. The long-read length is important for accurately identify transcript for non-model plant species with scarce molecular information such as the *Olea europaea*.

## Materials and Methods

### Plant material, salinity treatment, RNA extraction

Growth and salinity treatment of *Olea europaea* L. cv. Kalamon trees was performed as previously described [10] excluding the post-stress period. Four young trees from each treatment were used to collect tissues from roots and leaves after 90 days of treatment [10]. The tissues were washed repeatedly with deionised water, sterilized with 0.5% sodium hypochloride and ground with liquid nitrogen. For each tissue, total RNA was extracted according to the method of [28] after pooling of the four samples [10] and concentrated using the NucleoSpin H RNA Clean-up XS kit (Macherey-Nagel, Dueren, Germany). The RNA was quantified using a spectrophotometer and quality control was performed with Agilent 2100 Bioanalyzer.

### cDNA synthesis and 454 pyrosequencing

The cDNA library was constructed using a modification of the cDNA Rapid Library Preparation Kit (Roche Hellas). In brief, 2 μg total RNA from each sample was fragmented by adding 2 μl fragmentation solution in a total volume of 18 μl, vortexed and incubated at 72°C for 30 s. Precipitation was performed at -20°C overnight in a total volume of 500 μl with 50 μl NaOAc, 2.5 ml EtOH and 1 μl glycogen (Invitrogen/VWR, Tromsø, Norway). First strand cDNA was synthesized using 5’-TTTTTTCTTGTTTTCTTTTCTTV-3’ primer [29]. Second strand synthesis as well as library preparation was constructed following the instructions of GSFLX cDNA rapid library protocol. Library quantification and quality control was performed using Quantiflur ST Fluorometer (SB Biotechnology Suppliers S.A.) and Agilent 2100 Bioanalyzer respectively. Next generation sequencing was performed according to GSFLX Titanium protocols.

Raw sequences have been submitted to Sequence Read Archive (SRA) division of the Genbank repository and can be access through the SRA web site under accession number SRX297084, SRX297965, SRX297966, SRX297967.

### Vector removal

The obtained sequences were screened to remove vector contamination (trimming) using a simple ends free sequence alignment algorithm, cutadapt v.0.9 [30].

### CD-HIT-EST clustering

Following trimming, sequences were pooled in two datasets (a) roots treated and untreated and (b) leaves treated and untreated. Sequences in each dataset were clustered using CD-HIT-EST software [31] at a 95% similarity cut-off and sequence length ≥100 nucleotides. Algorithm parameters were set so as to compare both strands and assign the clustered sequences to the more similar cluster rather than the first cluster that meets the threshold.

### Differences in transcript abundance

The likelihood ratio R-statistic [32] was applied to each library pool to calculate the extent to which the differences in gene expression are due to a genuine biological effect and not due to non-biological sampling errors. The number of libraries (m) was assigned as m = 2 for each leaves and roots library.

### SSR identification

Mononucleotide-to-hexanucleotide Simple Sequence Repeats (SSRs) were identified using MISA (MIcroSAtellite identification tool) [33], downloaded from (http://pgrc.ipk-gatersleben.de/misa/). The parameters included a minimum of 10 mononucleotide repeats, 6 dinucleotide repeats, and 5 trinucleotide, tetranucleotide, pentanucleotide, and hexanucleotide repeats. The maximum number interrupting 2 SSRs in a compound microsatellite was set to 100bp (S1 Table).

### Homology search and Gene Ontology (GO) annotation

GO terms were assigned after blastx search of the sequences using Blast2GO software [34]. Threshold cut-off was at E-value 1.0e-6 and the alignment length of 33 amino acids.

### Quantitative real-time PCR analysis

Approximately 1 μg of RNA was reverse-transcribed using SuperScript™ II RNase H− Reverse Transcriptase (Invitrogen, Carlsbad, CA) and cDNA synthesis was performed according to manufacturer’s instructions, using Oligo dT^12–18^ primer.

Gene expression was determined using real-time PCR on a StepOne^TM^ Real-Time PCR system (Applied Biosystems, Foster City, CA). Total RNA was reverse transcribed as described above and the cDNA was used as template for real-time PCR. The PCR reaction (10 μl) mix consisted of gene specific primers, Maxima SYBR green/ROX qPCR Master Mix (Thermo Scientific, Waltham, MA) and the template. A first denaturation step at 95°C for 10 min was followed by 40 cycles each including a denaturation at 95°C for 15 s, an annealing at 60°C for 15 s and an extension step at 72°C for 15 s. A melting curve analysis protocol was executed in the temperature range from 60 to 95°C. The primers (S2 Table) were designed using the PRIMER3 software (Whitehead Institute for Biomedical Research, www-genome.wi.mit.edu/cgi-bin/primer/primer3.cgi/) while the GAPDH2 and PP2A1 were used as reference genes [35]. The qPCR was repeated twice. Data were analyzed using the 2−ΔΔCT method [36] and presented as relative levels of gene expression. The geometric mean of the two reference genes was used for normalization.

## Results

### Sequencing efficiency and specificity

In this study, we introduced the step of total RNA fragmentation instead of the double stranded cDNA fragmentation described by [19]. After the RNA fragmentation, the cDNA was synthesised using poly(T) primers which were designed (see Materials and Methods) in such a way in order to avoid the high light intensity produced through pyrosequencing of poly(A^+^) tails of short fragments. After ligation of the adaptors, the libraries were used in emulsion-based clonal amplification avoiding a cloning step in order to avoid bias. The sequencing was performed using a specific to adaptor primer. This way only poly(A^+^) tails comprising mRNAs were used as templates for cDNA synthesis suggesting sequencing of only one fragment per transcript.

### Raw and trimmed sequencing

Four cDNA collections were obtained comprising of 63,562–83,337 reads and a total number of 106.5 Mbp. These included cDNA of roots and leaves under salt and control conditions (Table 1). The length of raw reads was 332–388 nucleotides (nt). Trimming of raw reads caused a slight shortening resulting in a length of 329–387 nt and a total of 105.7 Mbp for all libraries (Table 1). The 454 raw data was processed following the workflow in S1 Fig.

**Table 1:** Sequencing data of the four cDNA libraries.

	Number of reads	Number of reads in tissue	Raw read length	Trimmed read length	Nucleotides in raw sequences	Nucleotides in trimmed sequences
Root salt-treated	74,777	158,114	346.98	344.77		
Root salt-untreated	83,337		388.54	386.50		
Leaves salt-treated	63,562	133,844	332.14	328.98		
Leaves salt-untreated	70,282		380.32	377.53		
Total	291,958	291,958	361.99	359.42	106,461,898	105,726,211

Number of reads: the number of sequenced reads in each cDNA library; Number of reads/tissue: the total number of sequenced reads in each tissue; Raw read length: the average length of raw reads; Trimmed read length: the average length of trimmed reads; Nucleotides in raw sequences: the total number of nucleotides in the raw sequences; Nucleotides in trimmed sequences: the total number of nucleotides in the trimmed sequences.

### Clustering and annotation of sequences in Roots and Leaves

**Fig 1:**
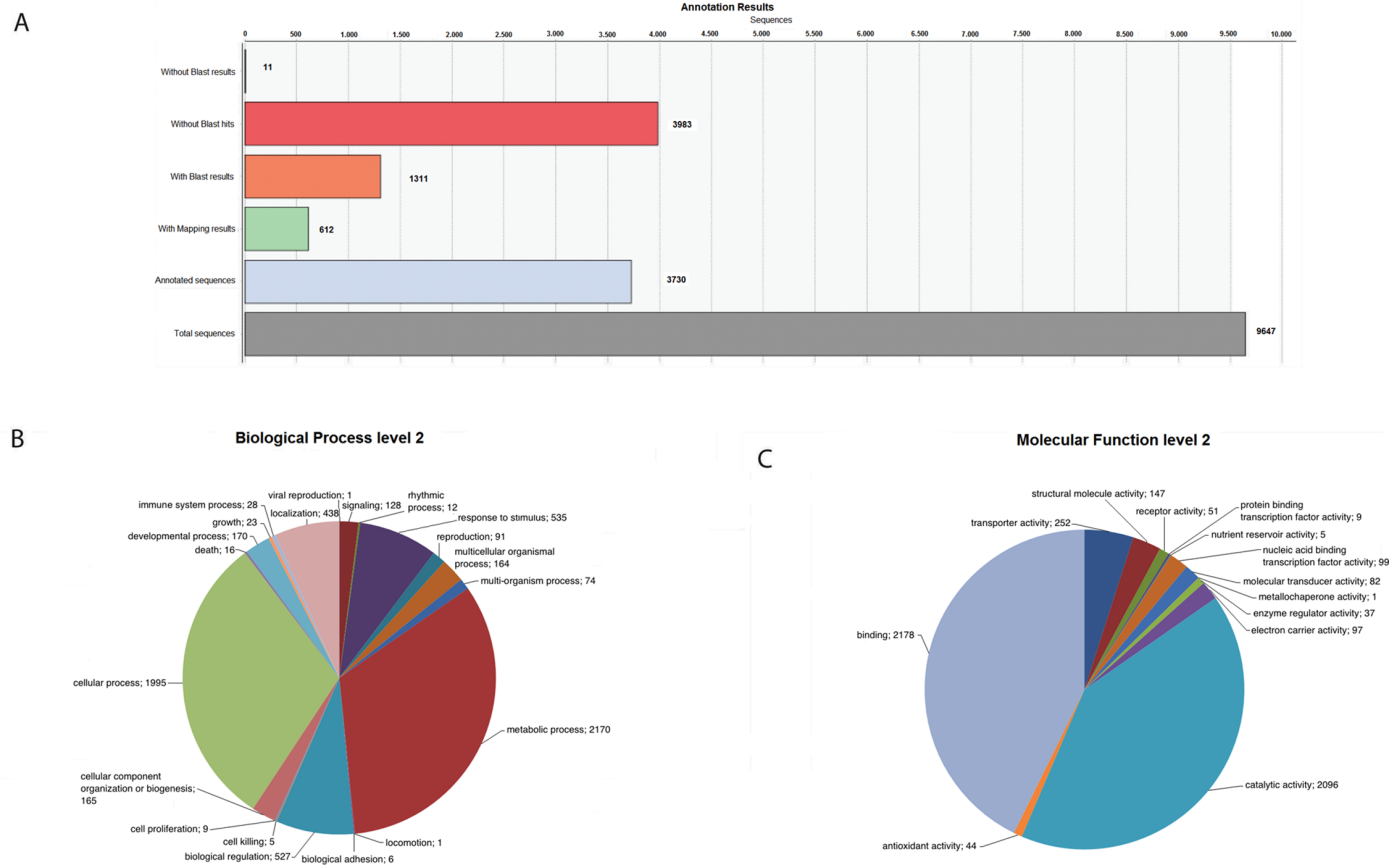
Annotation of 9,647 unique transcripts derived from roots’ cDNA libraries. A) Statistics of BlastX-based annotation results; B) Gene Ontology annotation related to biological processes; C) Gene Ontology annotation related to molecular functions.

**Fig 2:**
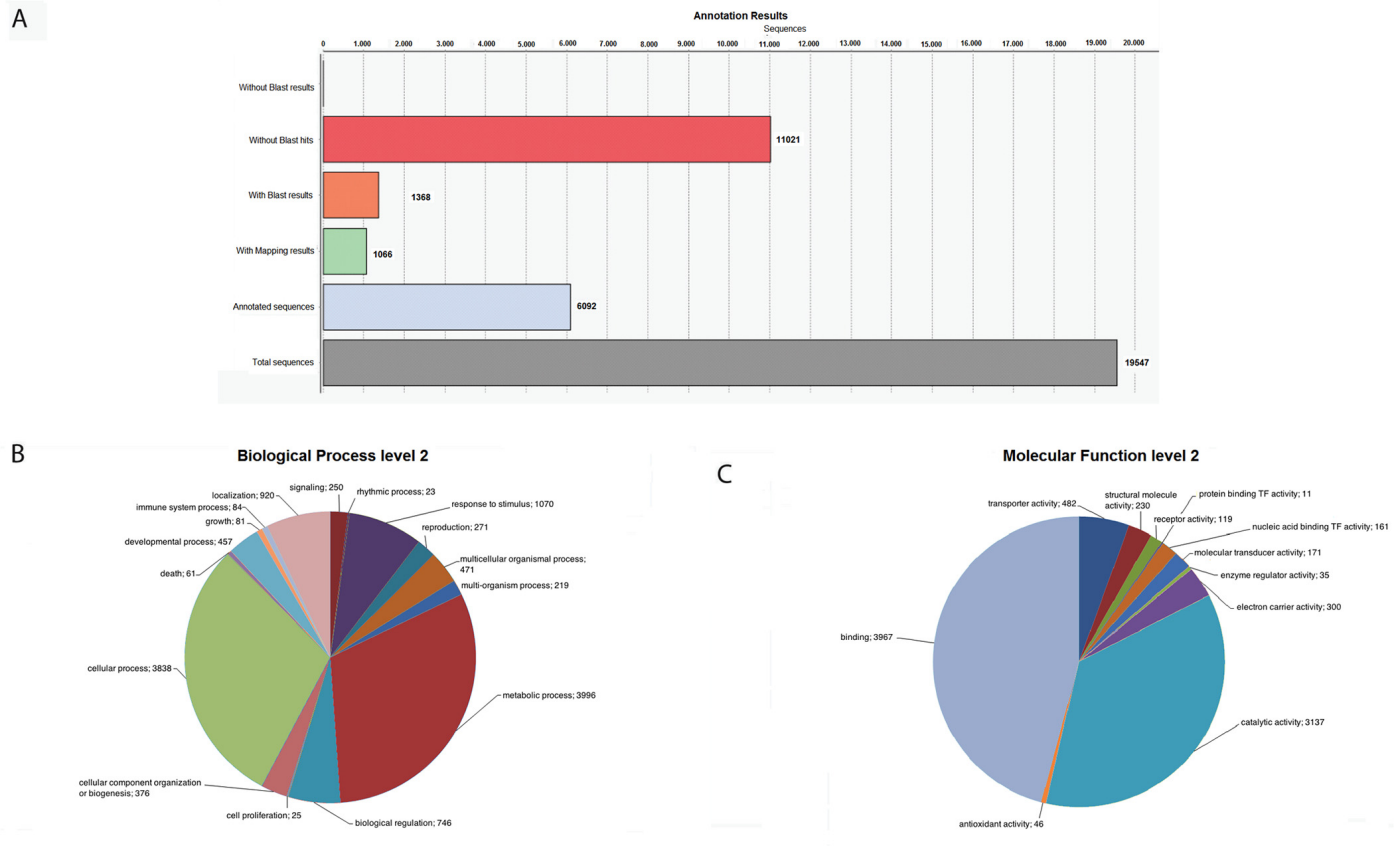
Annotation of 19,547 unique transcripts derived from leaves’ cDNA libraries. A) Statistics of BlastX-based annotation results; B) Gene Ontology annotation related to biological processes; C) Gene Ontology annotation related to molecular functions.

The reads of roots and leaves were initially treated as two distinct datasets comprising 158,114 and 133,844 reads, respectively (Table 1). Clustering of reads in each dataset resulted in the identification of clusters, representing unique transcripts. Each cluster can comprise more than 2 reads or appear as singleton (Table 2). In roots, clustering resulted in the identification of 9,647 clusters of which 8,023 are singletons (S3 Table). In leaves, clustering resulted in the identification of 19,547 clusters of which 2,642 comprise more than one read and 16,905 are singletons (S4 Table). BlastX-based annotation of each unique transcript resulted in 3,983 (41%) and 6,092 (31%) unique transcripts with significant similarities to proteins with known function in root and leaves, respectively (Fig 1A and Fig 2A).

**Table 2:** Clustering data.

	Total number of clusters	Number of clusters that comprise >2 reads	Number of singletons
Root	9,647	1,624	8,023
Leaves	19,547	2,642	16,905
Root and Leaves	28,270	4,210	24,060

Root: results of clustering in roots; Leaves: results of clustering in leaves; Roots and Leaves: results of clustering in the concatenated Roots and Leaves data.

The GO terms assigned to the transcripts of both tissues are mainly related to metabolism and include primary, cellular, macromolecule and nitrogen compound metabolic processes. Other prevailing annotations include terms related to biological regulation such as ‘regulation of biological process’ and ‘regulation of biological quality’, cellular process such as ‘cellular component organization’, response to stimulus, such as ‘response to chemical stimulus’, ‘response to stress’ and ‘response to abiotic stimulus’ and terms related to localization such as ‘establishment of localization’, ‘cellular localization’ and ‘macromolecule localization’ (Fig 1B and Fig 2B). Moreover the biological processes of ‘viral reproduction’, ‘locomotion’, ‘biological adhesion’ and ‘cell killing’ were annotated only at root transcripts.

The GO terms that describe the molecular functions of these transcripts include mostly ‘ion binding’, ‘transferase activity’, ‘nucleic acid binding’, ‘oxidoreductase activity’, ‘protein binding’ and ‘substrate-specific transporter activity’ (Fig 1C and Fig 2C). Comparing the molecular functions on olive root and leaf tissue, all the functions are commonly appeared in both tissues except 'metallochaperone activity' which is available only at root tissue. Metallochaperones are related with trafficking of metal ions within cells.

### Clustering and annotation of all sequences

The whole dataset of roots and leaves reads, comprising 291,958 transcripts, was clustered using sequencing similarity criteria leading to the identification of 4,210 clusters of at least 2 transcripts, and 24,060 singletons. The total number of clusters and singletons are referred to as unique transcripts.

### Transcripts encoding transcription factors

**Fig 3:**
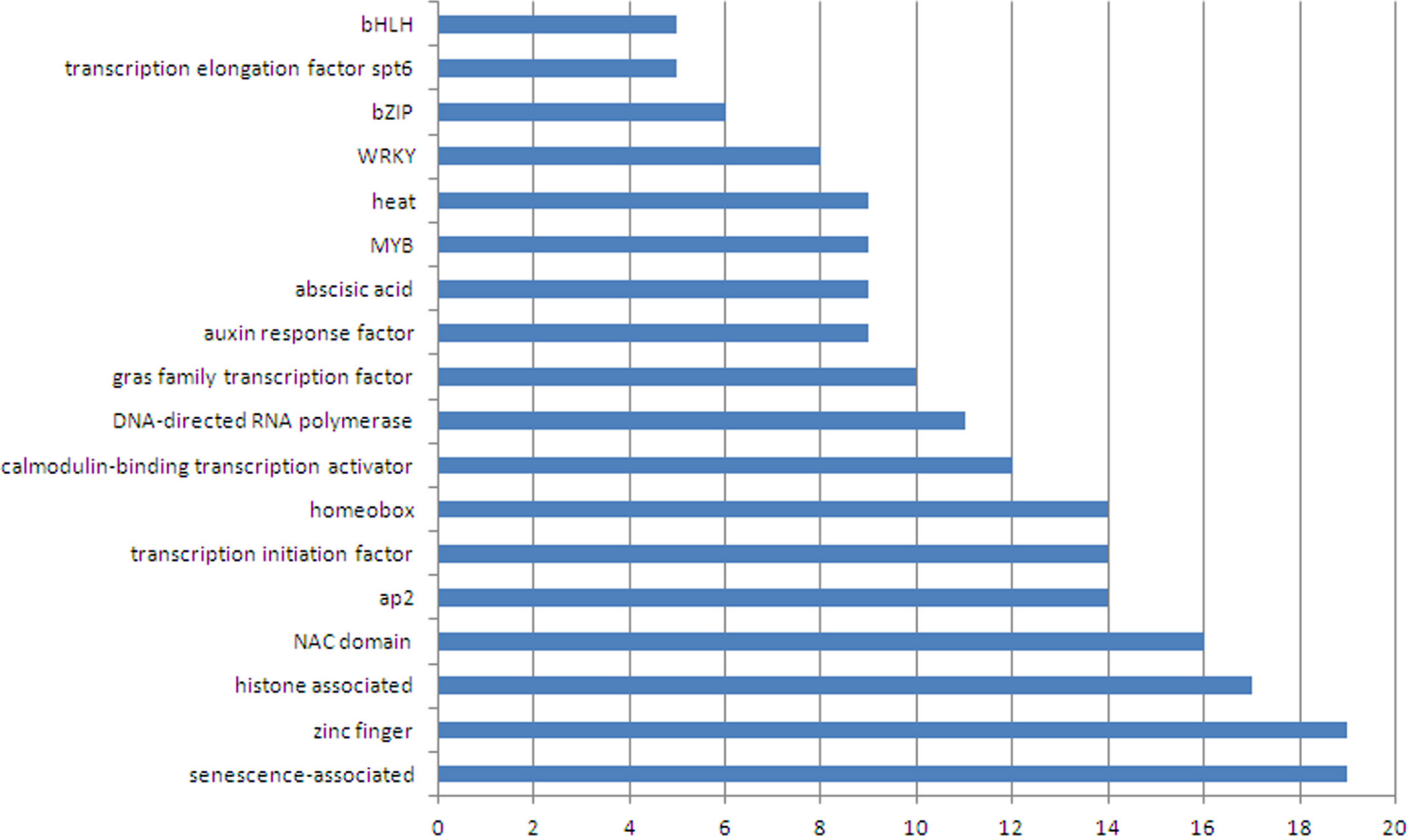
The most abundant transcription factors of olive root and leaves data. The x-axis shows the number of ESTs that are annotated on each group of TFs.

In this study 265 unique transcripts in roots and 433 unique transcripts in leaves were identified as transcription factors (TFs) or having transcription-related activity. The most abundant TFs appear to be senescence-associated TFs, zinc finger transcriptional regulators; histone associated transcriptional regulators, NAC domain TFs, AP2 and transcription initiation TFs, homeobox-related transcriptional regulators and calmodulin-binding transcriptional activators (Fig 3). The group of senescence-associated transcription factors comprises 19 members and represents the largest group of TFs indicating activation of the senescence process (Fig 3). Moreover, the NAC domain family of TFs which is also highly represented by 16 members is known to be involved in salt-stress-promoted senescence.

Among the TFs there was one JERF TF homologue (GRNLHQF14INXHL) which is identical to the jasmonic ethylene responsive factor (JERF) (se024_H1) known to be up-regulated after 45 days of salinity in cv. Kalamon [10]. In addition, a GRAS TF homologue (GRNLHQF16JXZTH) was identified to be identical to the GRAS (Olea_se015_B12) which is related to stimulus-response terms according to Blast2GO annotation and was part of a hierarchical TF network [10]. A third TF, a high-mobility group (HMG) homologue (GRNLHQF09FN48K) is identical to the HMG (Olea_cDNA_se024_H12) which is also part of a hierarchical TF network [10]. These results indicate that three TFs which are members of the regulatory networks identified in cv Kalamon in response to salinity [10] using a microarray approach were also identified to be expressed under salinity stress using a 454 pyrosequencing approach.

Other noteworthy annotated TFs are DNA-directed RNA polymerase transcriptional regulators, GRAS family transcription factors, auxin response transcription factors, abscisic acid related transcription factors, MYB transcription factor, heat-stress and heat shock related transcription factors, WRKY transcription factors, bZIP transcription factors, transcription elongation factor SPT6 and bHLH transcription factors (Fig 3).

### Salt stress related transcripts

The blastX-based annotation data was explored for the GO term ‘response to salt stress’ leading to the identification of 143 unique transcripts. Among them, there are transcripts such as aldehyde dehydrogenase, S-adenosyl methionine synthetase, aldoketoreductase, cinnamoyl reductase, malate oxidoreductase, isocitrate dehydrogenase, calcium-dependent protein kinase and monodehydro ascorbate reductase. This group comprises also transcripts related to salt tolerance such as the COBRA-like protein, kinase family protein, heat shock protein and abscisic acid-insensitive 5-like protein [37] (Table 3).

**Table 3:** Olive salt stress related transcripts.

Olive Sequencing ID	BlastX homology	Plant	GO annotation	Bibliography
GRNLHQF 09FM5BP	betaine aldehyde dehydrogenase	*Populus trichocarpa*	F:3-chloroallyl aldehyde dehydrogenase activity; P:response to water deprivation; F:aldehyde dehydrogenase (NAD) activity; C:peroxisome; P:oxidation reduction; P:response to abscisic acid stimulus; C:cytosol; F:betaine-aldehyde dehydrogenase activity; F:nucleotide binding	[38]
GRNLHQF 12HCONU	chlorophyll a b-binding	*Sorghum bicolor*	C:chloroplast stromal thylakoid; C:plastoglobule; P:photosynthesis, light harvesting; P:response to blue light; F:chlorophyll binding; P:nonphotochemical quenching; C:PSII associated light-harvesting complex II; P:response to far red light; P:response to red light; C:photosystem II antenna complex	[39–41]
GRNLHQF 12HC5WA	glutathione reductase	*Populus trichocarpa*	P:response to oxidative stress; P:gamete generation; C:cytosol; C:peroxisome; P:response to ionizing radiation; P:meiotic DNA double-strand break processing; P:cell redox homeostasis; P:oxidation reduction; P:glutathione metabolic process; F:glutathione-disulfide reductase activity; F:phosphogluconate dehydrogenase (decarboxylating) activity; F:3-hydroxybutyrate dehydrogenase activity; F:FAD binding; F:NADP or NADPH binding	[37]
GRNLHQF 12HDTPT	myo-inositol-1-phosphate synthase	*Arabidopsis thaliana*	C:cytoplasm; F:binding; P:phospholipid biosynthetic process; F:inositol-3-phosphate synthase activity; P:inositol biosynthetic process	[37]
GRNLHQF 11GW49N	proline dehydrogenase	*Raphanus sativus*	P:glutamate biosynthetic process; P:response to water deprivation; F:1-pyrroline-5-carboxylate dehydrogenase activity; P:proline catabolic process; P:response to osmotic stress; P:oxidation reduction; F:proline dehydrogenase activity; C:mitochondrion	[40]
GRNLHQF 10F3L4F	Salt Overly Sensitive 1	*Vitis vinifera*	C:integral to membrane; P:metabolic process; P:sodium ion transport; F:catalytic activity; P:transmembrane transport; F:solute:hydrogen antiporter activity	[42]
GRNLHQF 13HZDE5	superoxide dismutase	*Solanum lycopersicum*	C:chloroplast envelope; P:circadian rhythm; P:response to copper ion; F:copper ion binding; P:response to cadmium ion; C:plasma membrane; P:removal of superoxide radicals; F:superoxide dismutase activity; C:chloroplast stroma; C:thylakoid; C:cytosol; C:mitochondrion	[37]
GRNLHQF 09FIDP5	trehalose-6-phosphate synthase	*Nicotiana tabacum*	F:alpha,alpha-trehalose-phosphate synthase (UDP-forming) activity; C:cytosol; F:trehalose-phosphatase activity; P:trehalose biosynthetic process	[43]

The transcripts that were identified in roots and leaves and share homology with genes that have been previously identified as significant in salt stress response (E-value ≤ 1e^-6^).

**Fig 4:**
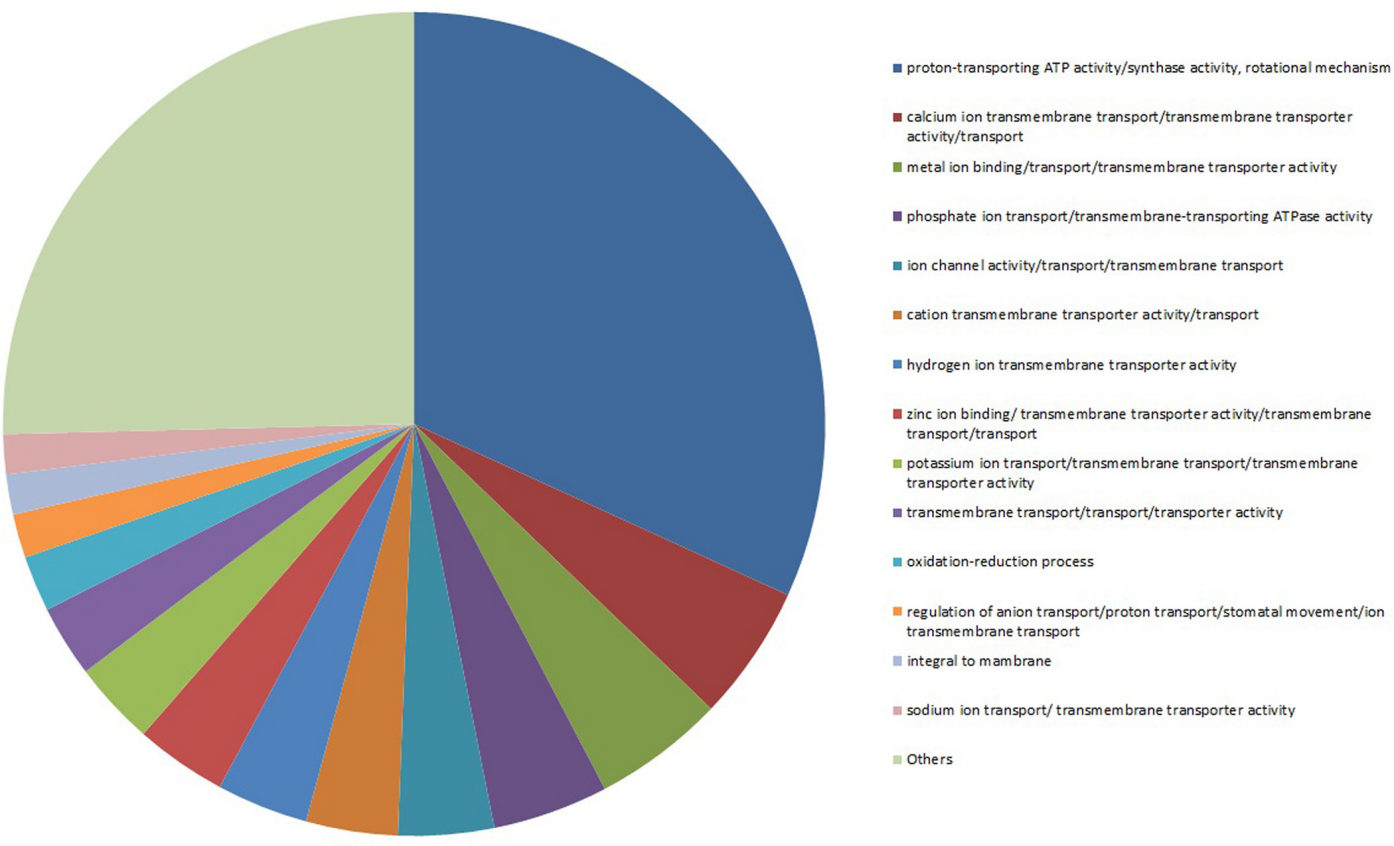
Transcripts related to ion transport. Pie chart of 387 unique transcripts that were annotated as ion transporters according to Blast2GO database.

In addition, 387 unique transcripts were identified as ion transporters. These include 9 transcripts related to sodium ion transport, 30 transcripts related to potassium ion transport whereas the majority of the transcripts were related to proton transport mechanism (Fig 4). More specific ion transporter include the V-type H^+^-ATPase (09FL244), Na^+^/H^+^ antiporter (GRNLHQF10GBOGJ) and a plasma membrane antiporter Salt Overly Sensitive 1 (SOS1) (GRNLHQF10F3L4F) as well as solutes transporters such as an inositol transporter 1 (GRNLHQF11GZ1U1), sorbitol transporter (GRNLHQF10F55QE) and polyol transporters 5-like (GRNLHQF13HVSNC). Moreover, salt-tolerance-specific genes which encode enzymes involved in the synthesis of osmoprotectants such as a myo-inositol 1-phosphate synthase (GRNLHQF12HDTPT) [38], proline dehydrogenase (GRNLHQF11GW49N) [37] and betaine aldehyde dehydrogenase (GRNLHQF09FM5BP) were also identified [39].

### Differentially expressed transcripts in roots and leaves

**Fig 5:**
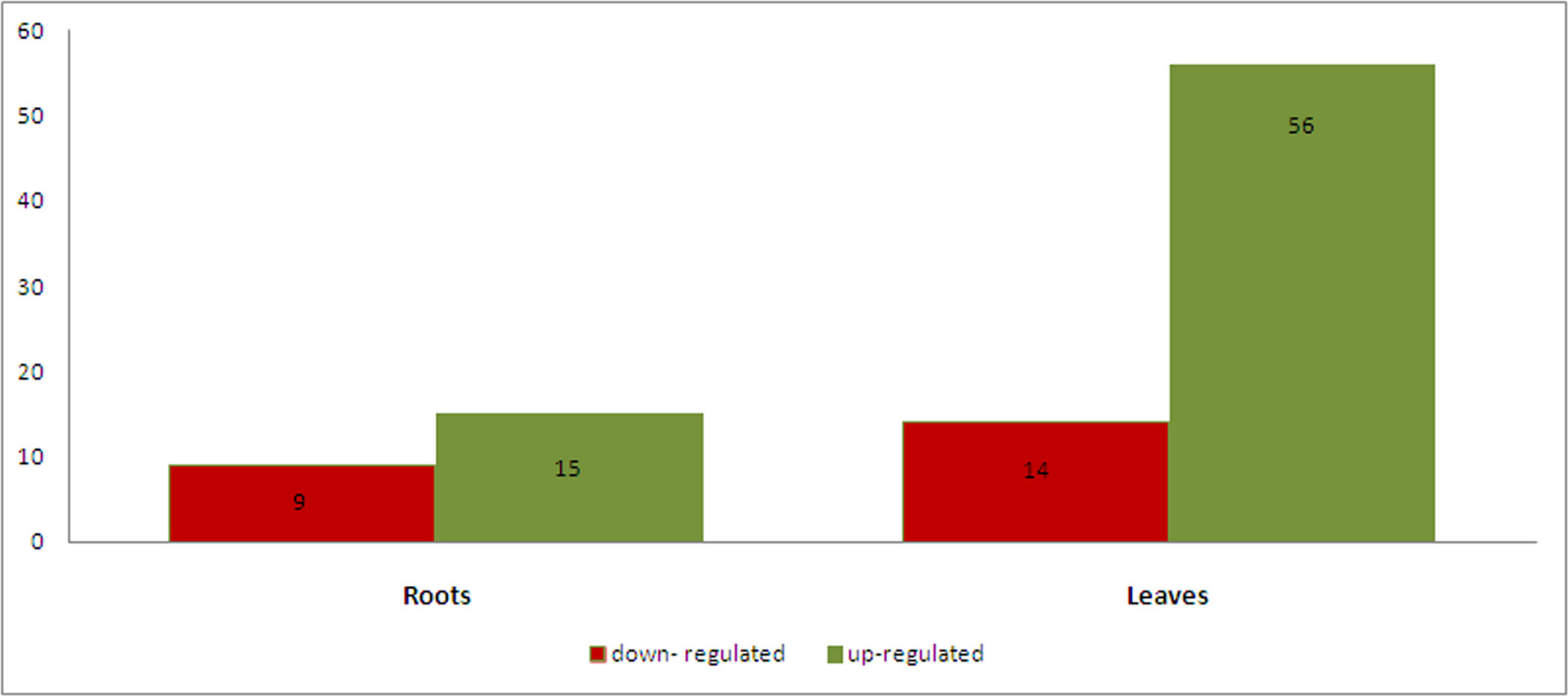
Differentially expressed transcripts in roots and leaves. In total, 24 and 70 genes in roots and leaves, respectively, were significantly differentially expressed under 90-day NaCl stress.

Transcripts present in roots and leaves were clustered according to their sequence similarity resulting in the identification of clusters (≥ 2 transcripts) and singletons (1 transcript). It is assumed that each cluster comprises transcripts of the same gene; therefore the number of transcripts in each cluster presumably corresponds to the expression level of the gene. Each cluster was classified as differentially expressed depending on the number of reads either in the NaCl-treated or untreated dataset. Among the 1,624 clusters in roots, the 24 were differentially expressed of which 9 were down- and 15 were up-regulated according to the statistical R-test. In leaves, among the 2,642 clusters, 70 were identified as differentially expressed, of which 14 were down- and 56 were up-regulated (Fig 5).

Abundant differentially expressed annotated clusters were identified as ‘senescence-associated’ in both roots (95.8% of the clusters) and leaves (64.3% of the clusters). Abundant differentially expressed clusters such as ‘ATP synthase subunit beta’ (83.3%), RNA intron encoded homing endonuclease (79.2%) and nuclear transcription factor Y subunit B18 (41.7%) were identified in roots and ‘cytochrome P450-like’ (60.0%), ‘ATP synthase subunit beta’ (57.1%) and ‘RNA intron encoded homing endonuclease’ (50.0%) in leaves. Other terms were also present to a lower extend as shown in Table 4.

**Table 4:** Significantly differentially expressed transcripts.

Term	Number of Root clusters	Number of Leaves cluster
senescence-associated	23 (95.8%)	45 (64.3%)
cytochrome P450-like	8	42 (60.0%)
ATP synthase subunit beta	20 (83.3%)	40 (57.1%)
rRNA intron-encoded homing endonuclease	19 (79.2%)	35 (50.0%)
nuclear transcription factor Y subunit B18	10 (41.7%)	15
Tar1p	2	7
Chk1 checkpoint-like kinase	3	6
tumor differentially expressed protein	1	4
10 kDa secreted protein		3
Ac1147-like partial		2
cell wall-associated hydrolase		2
cell wall-associated partial		2
novel Sal-like protein		2
PG1 protein		2
plasminogen		2
alternative oxidase		1
CG41536		1
conserved protein		1
leucine rich protein		1
lipoprotein		1
NADH-plastoquinoneoxidoreductase subunit-K		1
ORF64c [Arabidopsis lyrata subsp. lyrata]		1

The terms that characterize the transcripts in the clusters of Roots and Leaves with significantly differentially expressed transcripts.

**Fig 6:**
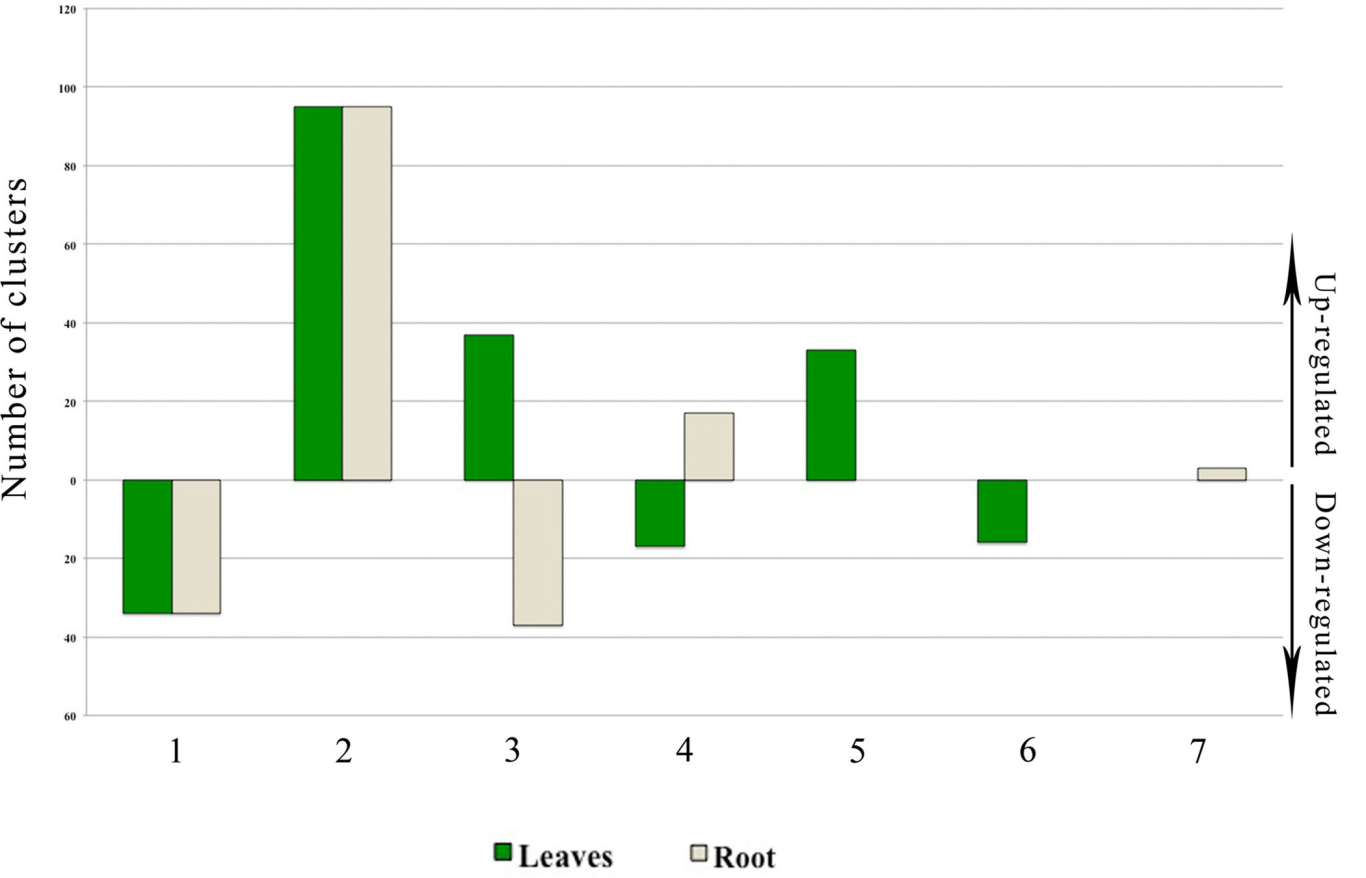
Differentially expressed transcripts in one or more tissue and/or treatment. 1) 34 common transcripts were down-regulated in both tissues; 2) 95 common transcripts were up-regulated in both tissues; 3) 37 common transcripts were up-regulated in leaves and down-regulated in root; 4) 17 common transcripts were down-regulated in leaves and up-regulated in root; 5) 33 transcripts were expressed and up-regulated only in leaves; 6) 16 transcripts were expressed and down-regulated only in leaves; 7) 3 transcripts were expressed and up-regulated only in root.

The R-test was applied to the entire dataset of the four libraries comprising 4,210 clusters leading to the identification of 235 differentially expressed clusters in one or more tissue and/or treatment (S5 Table). Among them, 34 and 95 were down- and up-regulated in both tissues, respectively (Fig 6). Furthermore, the expression of 37 transcripts was down-regulated in root but up-regulated in leaves while 17 transcripts showed exactly the opposite expression patterns. Interestingly, 49 transcripts in leaves and 3 in root showed tissue-specific expression.

**Fig 7:**
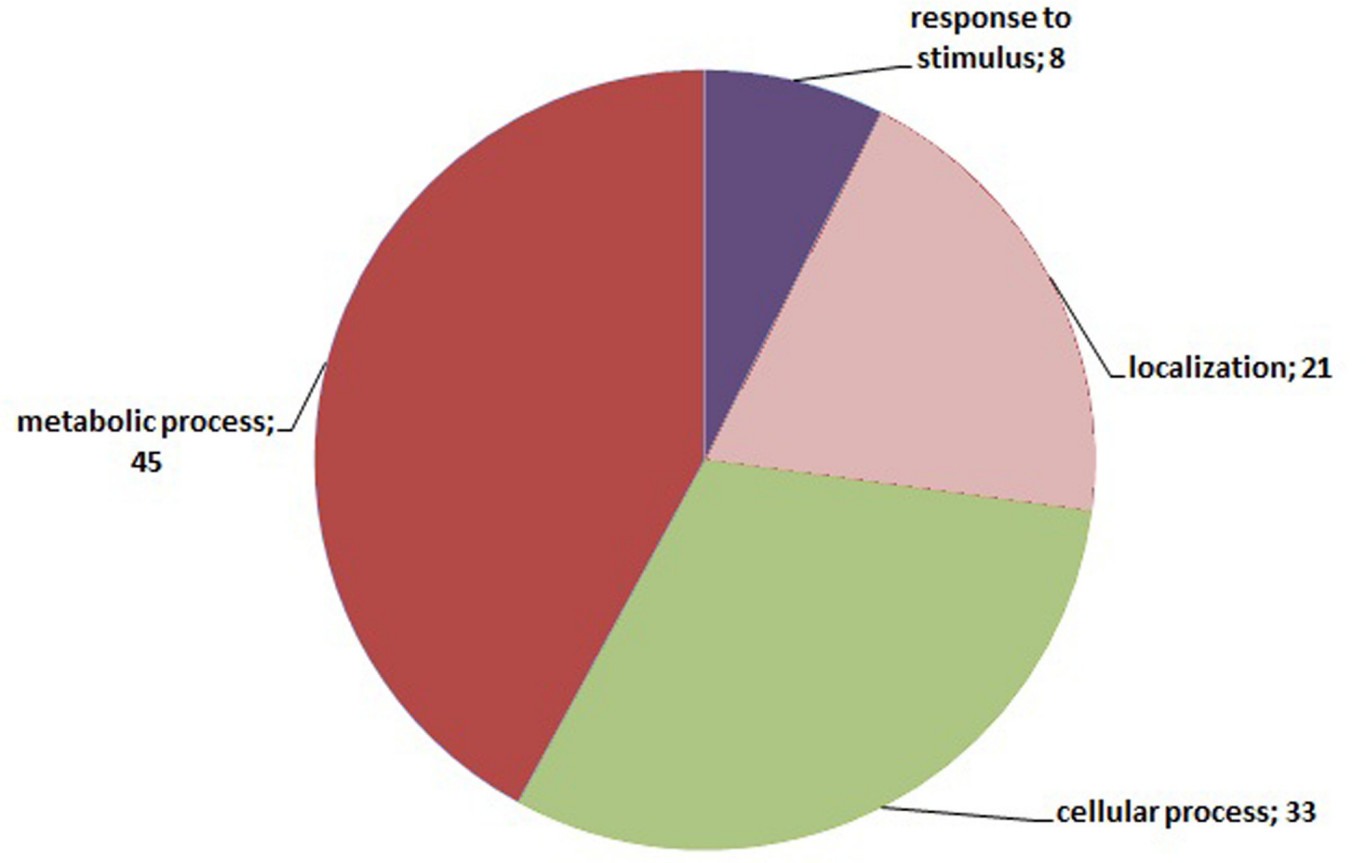
Gene Ontology annotation related to biological processes of the 235 differentially expressed clusters in one or more tissue and/or treatment derived from the entire dataset of the four libraries. Numbers in parenthesis show the number of times this GO term is present.

**Fig 8:**
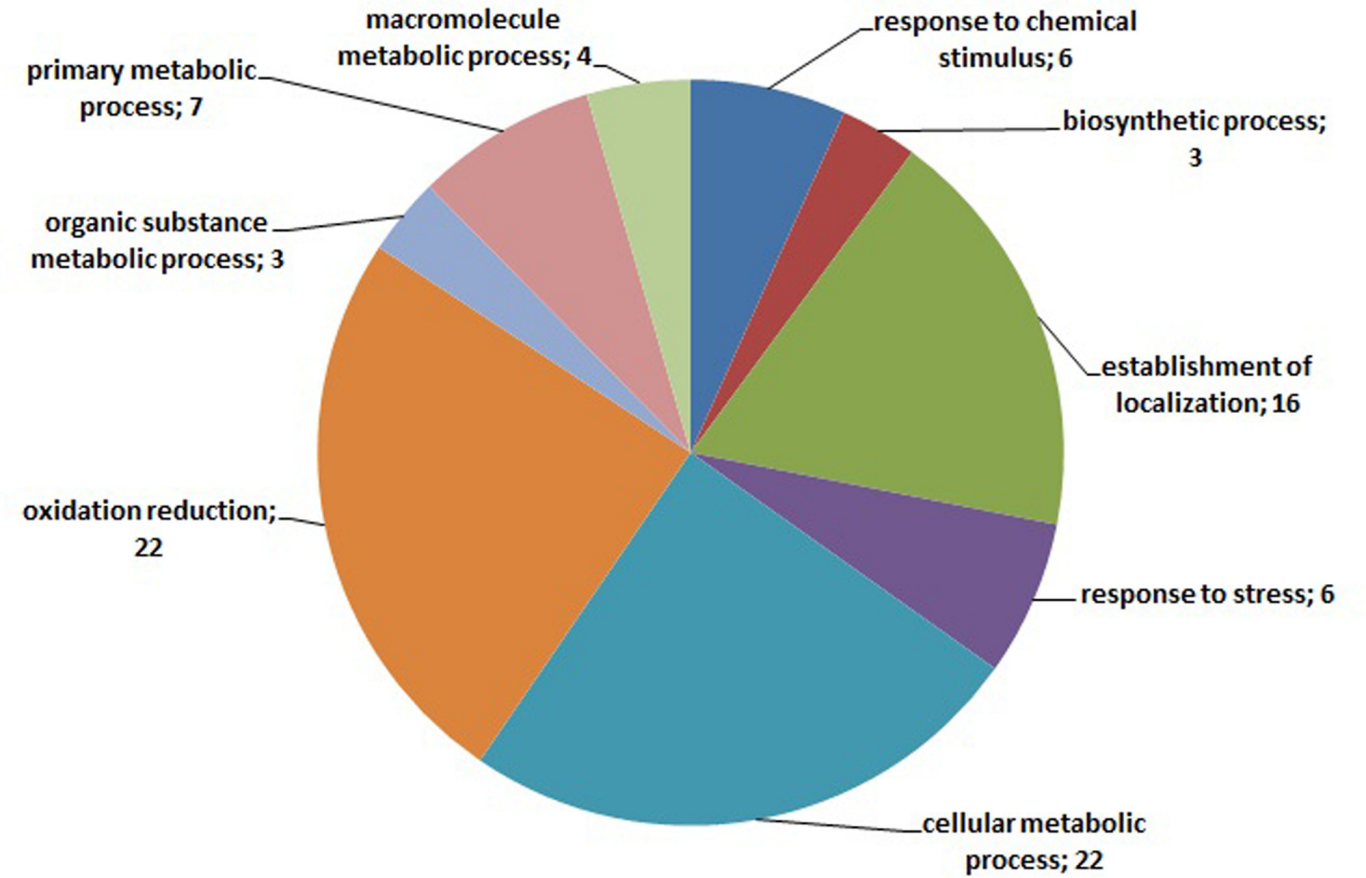
Gene Ontology annotation related to biological processes of the 49 leaf-specific differentially expressed clusters derived from the entire dataset of the four libraries. Numbers in parenthesis show the number of times this GO term is present.

**Fig 9:**
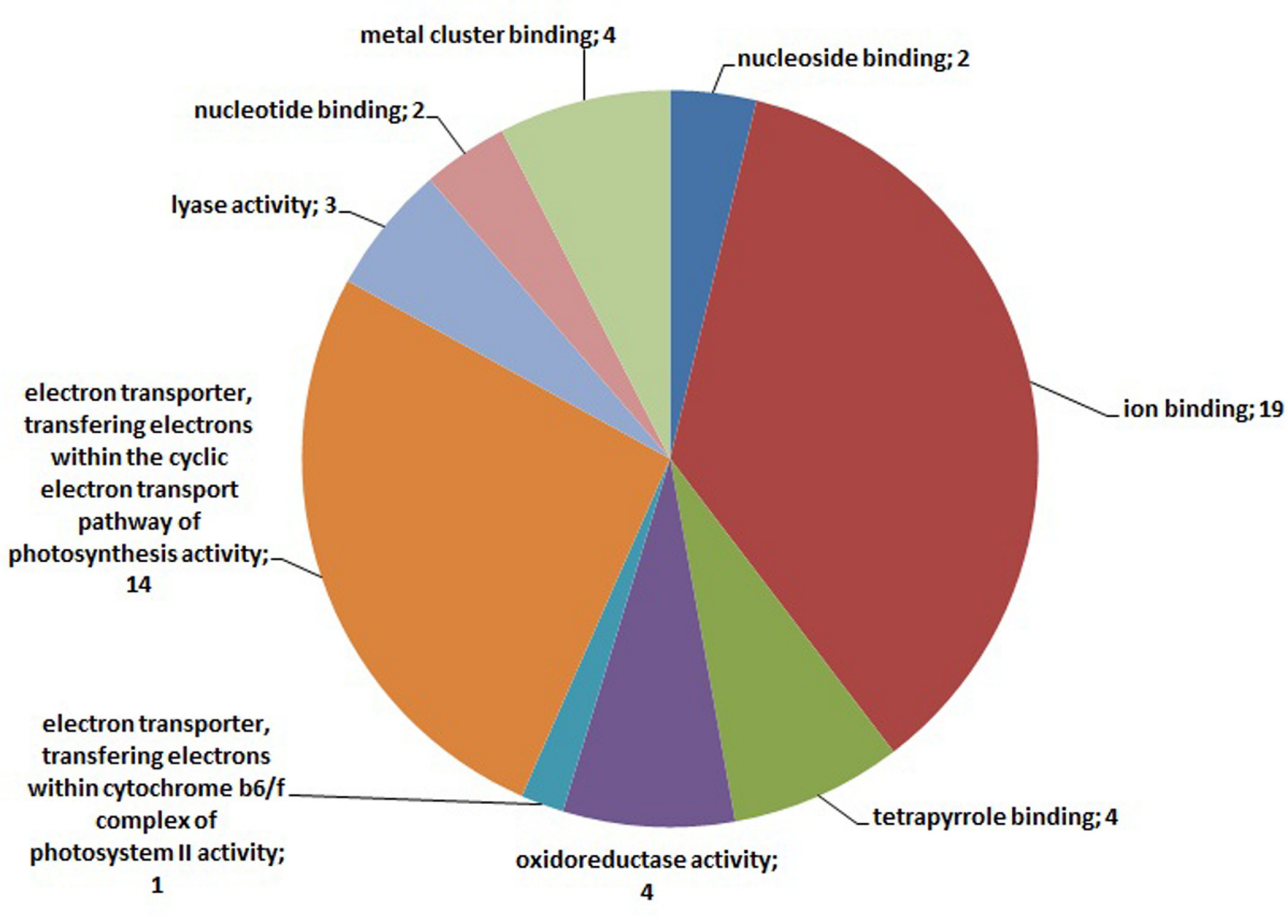
Gene Ontology annotation related to molecular functions of the 49 leaf-specific differentially expressed clusters derived from the entire dataset of the four libraries. Numbers in parenthesis show the number of times this GO term is present.

Annotation of the 235 transcripts indicates, mostly, terms related to ‘metabolic and cellular processes’ but it is noteworthy that 8 of them are related to ‘response to stimulus’ (Fig 7). Although no putative function was assigned for the 3 root-specific transcripts, the annotation of the 49 leaf-specific comprised many GO terms (Fig 8). These terms are mainly related to metabolic processes while 6 of the annotated transcripts are related to ‘response to stress’ (Fig 8). Regarding the leaf-specific transcripts with known molecular function, the GO terms that prevail are related to ‘ion binding’ and ‘photosynthesis activity’ (Fig 9).

### Real Time PCR analysis of NaCl-related transcripts

**Fig 10:**
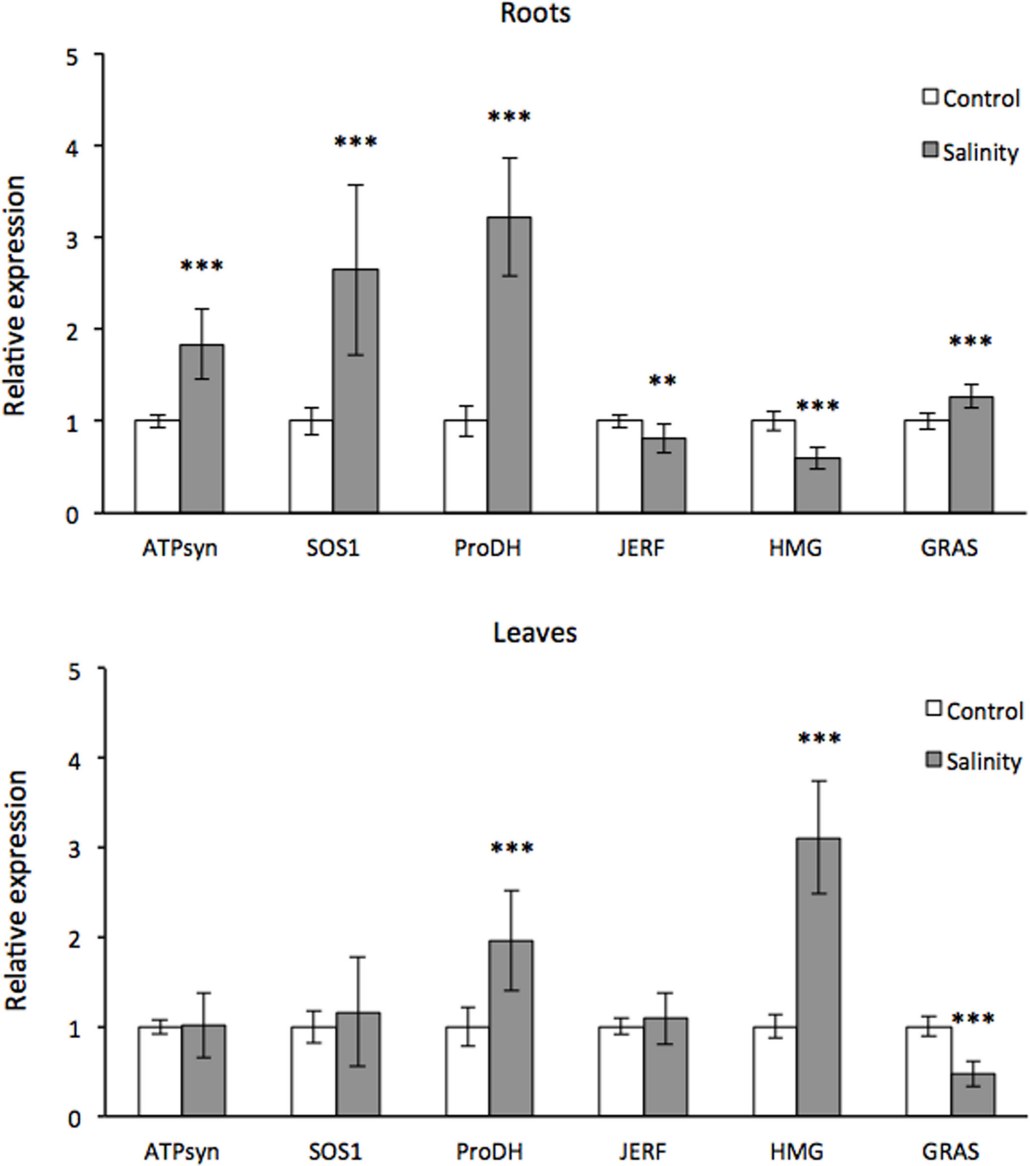
Expression levels of six NaCl-related transcripts. Bar graph for expression changes in 6 transcripts in both root and leaves determined by RT-qPCR. Each data point is the mean of two biological replications. Error bars represent the standard deviation of the means. Asterisks indicate statistically significant differences (*p<0.05, **p<0.01, ***p<0.001) as indicated by pairwise t-test between control and stress samples.

The expression levels of six transcripts comprising ATP synthase subunit beta, Salt Overly Sensitive 1 (SOS1), proline dehydrogenase and also three transcription factors such as the JERF, HMG and GRAS were determined (Fig 10). This way, the expression of these related to salinity genes was validated. The ATP synthase subunit beta was up-regulated almost by two fold in root but no change was observed in leaf while the transcript abundance of proline dehydrogenase increased in both root and leaf by three- and two-fold, respectively, validating the expression profiles that derived from RNA-seq (Fig 10). The SOS1 transcript increased by two and a half-fold in root and remained similar to control levels in leaf (Fig 10). The JERF transcription factor showed similar levels of expression in both root and leaf while the HMG transcription factor was down regulated by 50% in root but up-regulated by three-fold in leaf (Fig 10). The response of GRAS transcription factor to NaCl stress was marginal with a minimal up-regulation in root and down regulation by 50% in leaf (Fig 10).

## Discussion

The response of cv Kalamon in a 15-, 45- and 90-day NaCl treatment resulted in the identification of 209 differentially expressed transcripts; among them 159 were up-regulated throughout stress [10]. The remaining 50 were up-regulated only after 45 days of stress according to microarray analysis of a limited number of transcripts [10]. Although microarray analysis is an important technological platform for large-scale transcriptomic studies, there are several limitations such as the reduced accuracy of expression measurements for low abundance transcripts, the limited number of probes in non-model plants and the significant differences in the hybridization properties of probes [44].

Moreover, despite olive tree experiences mild salt stress in actual field conditions that causes yield losses, the transcriptome response of tolerant cultivars is poorly understood. Thus, the aim of the present study was to mimic olive growing field conditions under mild salt stress conditions, considering that cv. Kalamon may resist up to 200mM [45].

Based on the previous study [10], the 90-day time point was considered significant due to morphological and gene expression differences between a salt tolerant and salt sensitive cultivar. To further investigate in depth the response of cv Kalamon in salt stress, we monitored the transcriptional profile of roots and leaves of young olive trees in response to a 90-day NaCl (120 mM) treatment using 454 GS FLX pyrosequencing. This might be one of the first high-throughput transcriptome studies of olive under abiotic stress using this technological platform.

### Effectiveness of the approach

In our study the total RNA was fragmented prior to cDNA synthesis considering that fragments of a single cDNA might be cloned during library construction and sequenced leading to more than one reads per transcript providing wrong estimations on gene expression levels. In addition, construction of the cDNA libraries without cloning might avoid the bias considering that some cDNAs are potentially refractory to cloning [19]. This way, the quantification of gene expression should be considered reliable since the long reads ensures accurate transcript identification while sequencing of every transcript more than one time is avoided. These modifications might provide an improved approach for quantification of gene expression using NGS platforms even with lower depth of sequencing [46].

The issue of under-representation of long-size fragments caused by the inefficiency of the emulsion PCR for long PCR products might create a bias [19] which can be overcome by the random fragmentation-nebulization of cDNA fragments since it equally affects all transcripts [19]. Moreover tagging poly-A assures the one-read-one transcript relation and avoids assembly errors especially in light of paralogues genes, isoforms and alleles [19,29,46]. These techniques also indicate the reliability of the 454-GS FLX platform and the effectiveness of the approach, which used in this study [20–23, 25–26, 46–47].

### General transcriptome analysis and functional annotation

A total number of 28,270 unique transcripts was identified of which 35% are annotated, a percentage that is comparable to similar reports on non-model plants where about 70% of the transcripts did not have an assigned function [21, 25, 46]. The high percentage of transcripts with unknown function might correspond to tree species-specific genes which are quite divergent from other already annotated plant-model species [25]. The only tree species which has been extensively studied is *Populus trichocarpa*, which is evolutionary distant to *Olea europaea* [48]. Furthermore, a number of sequences may be related with non-coding RNAs, 5’ untranslated regions or sequences not containing a known protein domain [21].

The 9,822 annotated unigenes are involved in various biological processes, highlighting the potential of 454-GS FLX Titanium for transcriptome sequencing of non-model plants. Functional annotation of the transcripts revealed that when the terms are classified according to the biological process category, the majority are implicated in metabolic process, cellular process, and response to stimulus, localization and biological regulation. When the terms are classified according to the molecular function category, the prevailing terms are catalytic activity, binding and transporter activity. These results are in accordance with the prevailing terms that appear in NaCl-treated and NaCl-untreated unigenes of the halophytes *Reaumuria trigyna* and [49] and *Millettia pinnata* [50], both natural inhabitants of salinized soil.

### Ion transport and osmoprotectants

The low number of transcripts related to sodium and potassium ion transport might be explained by the low concentration of both ions in leaves due to sodium retention in the roots of salt tolerant cv Kalamon compared to other more sensitive to salinity cultivars [45]. It was suggested long time ago that differences in the capacity for sodium retention in roots reflects differences in salt tolerance [51]. Moreover, limited transcriptional response was also observed in studies that followed physiologically relevant mild abiotic stress treatments [52]. The low number of differentially expressed genes might indicate lower levels of gene expression activation probably due to the mild salt stress.

Among the ion transport transcripts, several salt-specific unigenes such as ion transporters and antiporters were annotated. The V-type H^+^-ATPase (GRNLHQF09FL244) is responsible for the active ion-transport to the vacuole using ATP hydrolysis [53] while its up-regulation is a well-known response of salt-tolerant plants to abiotic stress [54]. The Na^+^/H^+^ antiporter (GRNLHQF10GBOGJ) mediates the compartmentation of Na^+^ within the vacuole and its extrusion from the cell [55] and the plasma membrane antiporter Salt Overly Sensitive 1 (SOS1) (GRNLHQF10F3L4F) is known to efflux Na^+^ from Arabidopsis cells [43].

Moreover, the transcripts encoding three solutes transporters and three enzymes involved in the synthesis of osmoprotectants such as proline dehydrogenase might play an osmotic or protective role by preventing plant senescence, toxicity and high intercellular salt concentration and ensure plant survival through stress conditions [39, 56]. The up-regulation of three related to salinity genes such as SOS1, proline dehydrogenase and ATP-synthase beta-subunit mainly in roots after 90 days of NaCl treatment indicate the adaptive capacity of cv Kalamon to respond to this abiotic stress.

### Transcription factors

At the level of regulatory networks, the comparison of the interactions among TFs in olive with those reported for Arabidopsis might indicate similarities at the transcriptional level under salinity stress [10]. In this study a total of 71 unique transcripts were identified as having TF related functions. Among them, there are TFs known to be involved in ABA-mediated osmotic stress response such as WRKY, AP2/ERF, NAC, MYB, bHLH, NF-Y, HD-zip and bZIP while a MADS domain TF has recently been implicated in osmotic stress through ABA-mediated transcription [57].

Three TF homologues, JERF, HMG and GRAS, which were identified by 454 pyrosequencing, are also members of the hierarchical networks identified in cv. Kalamon in response to salinity using a microarray approach [10]. These results indicate that the same TF transcripts can be identified by using two different technological platforms confirming the validity of the 454 pyrosequencing data. The JERF homologue was not expressed in cv Chondrolia Chalkidikis during the stress period suggesting a significant difference of putative physiological significance between a salt tolerant and a salt sensitive cultivar [10]. Among the three transcription factors, only the HMG was significantly up-regulated while the other two showed marginal changes in transcript levels according to qPCR analysis. The high expression of the transcript that encodes HMG protein may indicate the significance of this TF in leaves.

It was also shown that 16 members of the NAC TF family were expressed after 90 days of salt stress while NAC transcripts are known to control a regulatory network during salt-promoted senescence in Arabidopsis [58] indicating similarities in the response of olive tree and Arabidopsis under salinity stress.

Leaf drop appeared only in cv Chondrolia Chalkidikis, the salt sensitive cultivar, but not in cv Kalamon after 90 days of salinity [10]. However, the “senescence-associated” and the NAC family of TFs were among the most abundant at this stage of stress suggesting active expression in leaves. This might indicate initiation of senescence although it has been reported absence of leaf drop in cv Kalamon after 150 days of 250 mM NaCl stress [45].

Although the WRKY transcription factors are the second most abundant TF family, they appear as singletons in our dataset. In roots all the WRKY transcripts were found in the untreated dataset while in leaves different singletons can be either in treated on in the untreated dataset consistent with the negative or positive role of different members of the WRKY family in plant signalling [59]. Specifically, the WRKY7 reduces its expression under salinity [60].

### ABA-related transcripts in roots and leaves

Moreover, in total, 18 (15 unique) ABA-related transcripts in roots and 170 (119 unique) in leaves were identified; a number which can be justified considering that ABA up-regulates gene expression under osmotic stress conditions [61–63]. In maize, although the ABA accumulation was increased up to 10-fold under salt stress in root tissues, which underlies the significant role of ABA in salt stress responses and/or adaptation [64–65].

### Comparison of the transcriptomes of roots and leaves upon salt stress

In olive trees, salinity above a critical level leads to stomatal closure, decrease in leaf area and reduction in shoot to root ratio [6]. This reduction might be attributed to the higher transcriptional activation observed in leaves compared to roots. Specifically, the number of clusters and singletons in leaves are more than double compared to roots while just the number of clusters is nearly 1.6 times more indicating higher transcriptional activation.

Furthermore, 70 differentially expressed clusters were identified in leaves compared to 24 in roots, a ratio which is higher compared to the one identified in maize in response to salt stress [66]. In maize, 206 genes were regulated specifically by the stress in leaves and 90 in roots [67]. Differentially expressed clusters in leaves but not in roots include terms related to photosynthesis (1 cluster), senescence (31 clusters), sodium channel and sodium ion transport (2 clusters). This indicates that roots and leaves differ considerably in their response to NaCl stress as shown in maize [66]. The majority of the differentially expressed annotated transcripts revealed relation to “senescence-associated” terms as was observed in chickpea [67] and maize [66].

The R-test was also applied to the entire dataset of the four libraries comprising of 4,210 clusters in order to identify differentially expressed tissue-related and/or tissue-specific transcripts. The clusters with differential expression in both tissues were 183.

BlastX-based analysis showed that 14 transcripts are related to cell wall associated hydrolases which are known to be involved in salt stress [68]. A decrease in hydrolases activity may cause decrease in wall elasticity, thus making them more rigid and tolerant under salt stress [68]. In our study 8 transcripts related with cell wall hydrolases are down regulated in root and only 1 in leaves.

Moreover, 19 clusters encode cytochrome P450s showing significant differential expression in leaves and roots. This large gene family is involved in biosynthesis of lignin [69] which plays important role in abiotic stress response, for long-distance water transport and during secondary cell wall formation [70].

Although the low depth of sequencing may be a limitation of this work since 85% approx. of clusters are singletons, the present study used oligo d(T) for poly(A) tagging, specially designed for 454 sequencing and thus the 3' UTR of each transcript was sequenced only once which increases the reliability of the present results even with lower sequencing depth. This method was also successfully applied in the European sea bass [46]. Furthermore, strict filtering criteria were used for clustering the sequences and the likelihood ratio R-statistic [32] was applied to calculate the extent to which the differences in gene expression are due to a genuine biological effect and not due to non-biological sampling errors. Consequently, the significant differentially expressed clusters contain at least 29 reads with the majority of them including more than 100 reads. Therefore these results might be considered robust providing an important tool for more advanced molecular studies in olive.

This 454-based high throughput transcriptome analysis resulted in a more thorough investigation of the Kalamon leaves and roots gene expression profile in response to mild salt stress considering that 9,822 unigenes were annotated compared to the 1,121 non-redundant ESTs which were analysed in the previous effort [10].

However a deep analysis has to be performed in future studies in order to characterize in a more detailed way the transcriptome under abiotic stress conditions.

## Supporting Information

S1 Fig 454-raw data processing work-flow for comparative transcriptome analysis. (TIF)

S1 Table Statistics of SSRs in Roots and Leaves of olive. (DOCX)

S2 Table Primers used for the real-time quantitative PCR. (DOCX)

S3 Table Expression changes of clusters across conditions resulted from clustering the ESTs of Roots. (XLSX)

S4 Table Expression changes of clusters across conditions resulted from clustering the ESTs of Leaves. (XLSX)

S5 Table Significant differentially expressed clusters across conditions resulted from clustering the ESTs of Roots and Leaves together. (XLSX)
