# Supplementary figures and images for: 454 Pyrosequencing of Olive (Olea europaea L.) Transcriptome in Response to Salinity
Source: PLoS One. 2015 Nov 17;10(11):e0143000. doi: 10.1371/journal.pone.0143000 (PMC4648586; doi:10.1371/journal.pone.0143000)

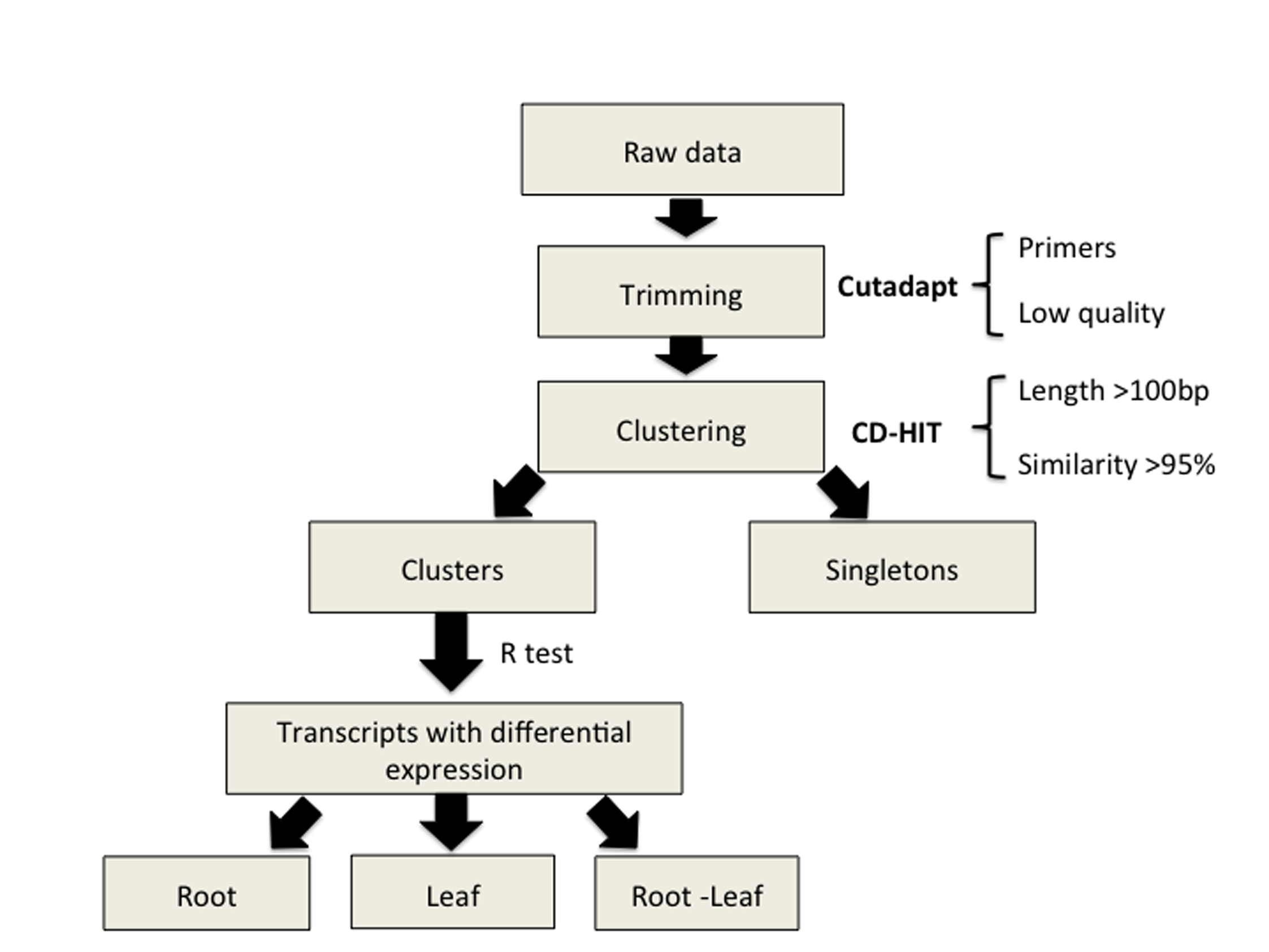

Supplement: S1 Fig — (TIF) [file pone.0143000.s001.tif]
