# Supplementary material for: 454 Pyrosequencing of Olive (Olea europaea L.) Transcriptome in Response to Salinity
Source: PLoS One. 2015 Nov 17;10(11):e0143000. doi: 10.1371/journal.pone.0143000 (PMC4648586; doi:10.1371/journal.pone.0143000)
Supplement: S1 Table — (DOCX) [file pone.0143000.s002.docx]

S1 Table. Statistics of SSRs in Roots and Leaves of olive.

|  | Roots | Leaves |
| --- | --- | --- |
| Number of sequences examined | 9647 | 19547 |
| Size of examined sequences | 3283509 bp | 6551787 bp |
| Number of identified SSRs | 978 | 1880 |
| Number of sequences containing SSRs | 557 (5.8%) | 1286 (6.6%) |
| Number of sequences containing more than one SSR | 134 | 228 |
| Mononucleotides | 780 | 1473 |
| Dinucleotides | 78 | 191 |
| Trinucleotides | 95 | 177 |
| Tetranucleotides | 8 | 19 |
| Pentanucleotides | 3 | 11 |
| Hexanucleotides | 14 | 9 |
