# Supplementary material for: 454 Pyrosequencing of Olive (Olea europaea L.) Transcriptome in Response to Salinity
Source: PLoS One. 2015 Nov 17;10(11):e0143000. doi: 10.1371/journal.pone.0143000 (PMC4648586; doi:10.1371/journal.pone.0143000)
Supplement: S2 Table — (DOCX) [file pone.0143000.s003.docx]

**S2 Table. Primers used for the real-time quantitative PCR**

| **Gene name** | **Accession Nr.** | **Gene description** | **Primer sequence (forward / reverse)** | **Product size** |
| --- | --- | --- | --- | --- |
| GAPDH2 | OLEEUCl022518\| Contig2 | glyceraldehyde-3-phosphate dehydrogenase | CCTTCCGTGTGCCTACTGTT  GATGGCTGCCTTGATTTCAT | 92 |
| PP2A1 | OLEEUCl021848\| Contig2 | serine/threonine-protein phosphatase 2A | TGCAGTGGCTACAGGACAAG  TGGACCAAATTCTTCAGCAA | 83 |
| ATPsyn | GRNLHQF09FL244 | ATP-synthase subunit | CACTGCTCAAATTGTTCTCCAATAG  GGAGACTTTCGGAATTGAAGACTA | 91 |
| SOS1 | GRNLHQF10F3L4F | salt overly sensitive 1 | GGATAAGCTAACAGCGGCTAAGA  CCAAGATCACCAAATGCCTCTA | 90 |
| ProDH | GRNLHQF11GW49N | proline dehydrogenase | GGCCTATTCAGGGAGTGGTAAA  CTACTGAGATGGCAATACAAGGAT | 107 |
| JERF | GRNLHQF14INXHL | ethylene response factor | GGTGGAGTTTACTGAGCACCATT  TGTCTTCTACAGCACCAAAGCAT | 109 |
| HMG | GRNLHQF09FN48K | high mobility group transcription factor | CAAGTCCAAAATCCGAAGTGAATG  CTCTCTACTCGTCGTCGTCATCT | 87 |
| GRAS | GRNLHQF16JXZTH | GRAS transcription factor | GGTCATGGTGGTTACTGAACAAG  GACTCCAAACTGTCAAACAATGC | 108 |
